# Potent interaction of flavopiridol with MRP1

**DOI:** 10.1038/sj.bjc.6690687

**Published:** 1999-09

**Authors:** J H Hooijberg, H J Broxterman, G L Scheffer, C Vrasdonk, M Heijn, M C de Jong, R J Scheper, J Lankelma, HM Pinedo

**Affiliations:** Departments of Medical Oncology and, Pathology, Academisch Ziekenhuis Vrije Universiteit, PO Box 7057, Amsterdam, 1007 MB, The Netherlands

**Keywords:** multidrug resistance, MRP1, (iso)flavonoids, flavopiridol, ATPase

## Abstract

The multidrug resistance protein 1 (MRP1) is an ATP-dependent transport protein for organic anions, as well as neutral or positively charged anticancer agents. In this study we show that flavopiridol, a synthetic flavonoid currently studied in phase 1 trials for its anti-proliferative characteristics, interacts with MRP1 in a potent way. Flavopiridol, as well as other (iso)flavonoids stimulate the ATPase activity of MRP1 in a dose-dependent way at low micromolar concentrations. A new specific monoclonal antibody against MRP1 (MIB6) inhibits the (iso)flavonoid-induced ATPase activity of plasma membrane vesicles prepared from the MRP1 overexpressing cell line GLC_4_/ADR. The accumulation of daunorubicin in GLC_4_/ADR cells is increased by flavopiridol and by other non-glycosylated (iso)flavonoids that interact with MRP1 ATPase activity. However, flavopiridol is the only tested compound that affects the daunorubicin accumulation when present at concentrations below 1 μM. Glycosylated (iso)flavonoids do not affect MRP1-mediated transport or ATPase activity. Finally, MRP1 overexpressing and transfected cells are resistant to flavopiridol, but not to other (iso)flavonoids tested. These findings may be of relevance for the development of anticancer therapies with flavopiridol. © 1999 Cancer Research Campaign


*British Journal of Cancer* (1999) **81**(2), 269–276
© 1999 Cancer Research Campaign
Article no. bjoc.1999.0687

### Potent interaction of flavopiridol with MRP1

**JH Hooijberg[1], HJ Broxterman[1], GL Scheffer[2], C Vrasdonk[1], M Heijn[1], MC de Jong[2], RJ Scheper[2], J Lankelma[1] and HM Pinedo[1]**

Departments of [1]Medical Oncology and [2]Pathology, PO Box 7057, Academisch Ziekenhuis Vrije Universiteit, 1007 MB Amsterdam, The Netherlands

**Summary** The multidrug resistance protein 1 (MRP1) is an ATP-dependent transport protein for organic anions, as well as neutral or positively charged anticancer agents. In this study we show that flavopiridol, a synthetic flavonoid currently studied in phase 1 trials for its anti-proliferative characteristics, interacts with MRP1 in a potent way. Flavopiridol, as well as other (iso)flavonoids stimulate the ATPase activity of MRP1 in a dose-dependent way at low micromolar concentrations. A new specific monoclonal antibody against MRP1 (MIB6) inhibits the (iso)flavonoid-induced ATPase activity of plasma membrane vesicles prepared from the MRP1 overexpressing cell line $GLC_4$/ADR. The accumulation of daunorubicin in $GLC_4$/ADR cells is increased by flavopiridol and by other non-glycosylated (iso)flavonoids that interact with MRP1 ATPase activity. However, flavopiridol is the only tested compound that affects the daunorubicin accumulation when present at concentrations below 1 μM. Glycosylated (iso)flavonoids do not affect MRP1-mediated transport or ATPase activity. Finally, MRP1 overexpressing and transfected cells are resistant to flavopiridol, but not to other (iso)flavonoids tested. These findings may be of relevance for the development of anticancer therapies with flavopiridol.

© 1999 Cancer Research Campaign

**Keywords:** multidrug resistance; MRP1; (iso)flavonoids; flavopiridol; ATPase

The emergence of resistance of tumour cells to chemotherapy is one of the obstacles for a more effective treatment of cancer patients. One form of cellular resistance, directed against a wide spectrum of natural agents, is referred to as multidrug resistance (MDR). Among others, the overexpression of drug transporter proteins, like the *MDR1* encoded P-glycoprotein (Pgp) (Gottesman et al, 1993) and the *MRP-1* encoded multidrug resistance protein 1 (MRP1) (Cole et al, 1992, 1998) can cause MDR. These proteins, which belong to the superfamily of ATP binding cassette (ABC) transporters (Higgins, 1992), can cause cellular resistance to agents such as the anthracyclines, the vinca alkaloids and taxol. Pgp and MRP1 appear to mediate the transport of a broad range of drugs across cellular membranes (Versantvoort et al, 1992; Gottesman et al, 1993). Pgp is thought to transport neutral or positively charged compounds, whereas MRP1 is a transporter of neutral and anionic compounds such as glutathione-conjugated drugs, sulphates and glucuronidates (Jedlitschky et al, 1994; Broxterman et al, 1995). Therefore, MRP1 is considered to be a multiple organic anion transporter (Jedlitschky et al, 1994; Müller et al, 1994).

The ATP-dependent transport of anticancer drugs out of tumour cells that express MDR proteins may at least theoretically lead to impaired treatment outcome of patients. Therefore, much effort has been directed at the identification of agents, which inhibit Pgp- or MRP1-mediated drug transport. Among others, several benzo-γ-pyrones, also called (iso)flavonoids, were found to be able to modulate Pgp- and MRP1-mediated drug transport in tumour cell lines (Critchfield et al, 1994; Hooijberg et al, 1997). Many (iso)flavonoids are present in our daily food, since they are metabolic products of plant species (Kuehnau, 1976). The interest in these compounds is based on their biochemical activities, such as phosphotyrosine kinase inhibition (Akiyama et al, 1987; Glossmann et al, 1981), topoisomerase II inhibition (Markovits et al, 1989), and scavenging of free radicals (Bors et al, 1987; Cotelle et al, 1992). Recently, the synthetic compound flavopiridol has drawn considerable attention for its antiproliferative capacities as an inhibitor of cyclin-dependent kinases cdk1, cdk2, cdk4 and cdk7 (Sedlacek et al, 1996). Several phase 1 studies with flavopiridol are currently in progress (Senderowicz et al, 1998; Stinson et al, 1998), while several (iso)flavonoids have been reported to act as inhibitors of angiogenesis, implicating a potential role in the prevention as well as the treatment of cancer (Fotsis et al, 1993).

In the past, the (iso)flavonoid genistein was shown to inhibit the efflux of daunorubicin (DNR) from the MRP1 overexpressing human small-cell lung cancer $GLC_4$/ADR cells in a competitive way (Versantvoort et al, 1994). In addition, we recently reported that the ATPase activity of plasma membranes prepared from $GLC_4$/ADR cells, but not of parental $GLC_4$ cells, was increased in the presence of high micromolar concentrations genistein and several other (iso)flavonoids, among which flavopiridol (Hooijberg et al, 1997).

In the present work we demonstrate that the specific induction of MRP1 ATPase activity by the flavonoid flavopiridol, and other non-glycosylated (iso)flavonoids, is dose-dependent at low micromolar concentrations. The effects of non-glycosylated (iso)flavonoids on MRP1 ATPase activity are paralleled by increasing effects on cellular DNR accumulation in intact MRP1 overexpressing cells. In particular, flavopiridol is shown to modulate the DNR accumulation already at low micromolar concentrations. In addition, MRP1 overexpressing cells are found to be resistant to flavopiridol.

*Received 8 December 1998*
*Revised 22 March 1999*
*Accepted 31 March 1999*

*Correspondence to:* HJ Broxterman

### MATERIALS AND METHODS

### Chemicals

[$^3$H]GSH (1.6 TBq mmol$^{-1}$) was from New England Nuclear (Dreieich, Germany). Dithiothreitol (Sigma, St Louis, MO, USA) was separated from [$^3$H]GSH according to Butler et al (1976). [$^3$H] dinitrophenyl-S-glutathione (DNP-SG) was synthesized as described earlier (Heijn et al, 1997). 1-Chloro-2,4-dinitrobenzene (CDNB) was from Sigma (St Louis, MO, USA). The flavonoid kaempferol, the isoflavonoid genistin, malachite green base, ammonium molybdate, Triton N101, probenecid and ATP were from Sigma (St Louis, MO, USA). The flavonoids apigenin, apigetrin and kaempferol-3-glucose were from Extrasynthese (Genay, France). The isoflavonoid genistein was from ICN Biomedicals (Zoetermeer, The Netherlands). The synthetic flavone derivative flavopiridol (L86-8275, NSC 649890) (–)cis-5,7-dihydroxy-2-(2-chlorophenyl)-8[4-(3-hydroxy-1-methyl)-piperidinyl]-4H-benzopyran-4-one was kindly provided by Dr Böhme (Hoechst Marion Roussel, Cincinnatti, OH, USA). The (iso)flavonoids were dissolved as a stock solution of 40 mM in dimethyl sulphoxide (DMSO; Across Chimica, Belgium) and stored at –20°C. Before experiments, 1:1 dilutions were made in ethanol, followed by a dilution in ethanol/water (1: 3; v/v) to the appropriate concentrations. The maximal concentration of DMSO and ethanol, which was also added to the controls, was 0.5% (v/v). Membrane filters OE67 were from Schleicher and Schuell (Dassel, Germany). Doxorubicin hydrochloride was from Laboratoire Roger Bellon (France). DNR hydrochloride was from Specia (Paris, France) and [$^3$H]DNR hydrochloride (163 GBq mmol$^{-1}$) from Dupont de Nemours (Den Bosch, The Netherlands).

### Cell lines

The human small-cell lung cancer cell line $GLC_4$ and its adriamycin-selected MRP1 overexpressing subline $GLC_4$/ADR (Zijlstra et al, 1987) were cultured in RPMI-1640 medium (Flow Labs, Irvine, UK), supplemented with 10% (v/v) heat-inactivated fetal calf serum (FCS; Gibco, Paisley, UK). $GLC_4$/ADR cells were cultured in the presence of 1.2 µM doxorubicin until 7–14 days before the experiments. The human acute myeloblastic leukaemia cell line HL-60 and its MRP1 overexpressing subline HL-60/ADR (Krishnamachary et al, 1994) were cultured as the $GLC_4$ cell line. HL-60/ADR cells were cultured in the presence of 0.35 µM doxorubicin, until 7–14 days before the experiments. The non-small-cell lung cancer cell line SW-1573 and the stable MRP1-transfected SW-1573 cell line S1(MRP1) and MDR1-transfected S1(1.1) have been described earlier (Zaman et al, 1995). S1(MRP1) cells were cultured in the presence of G418 until 2–10 days before experiments.

### Plasma membrane vesicles

Plasma membrane vesicles were prepared from parental $GLC_4$ and drug-resistant $GLC_4$/ADR cells as described (Heijn et al, 1997). Cells were harvested by centrifugation (275 ***g***, 5 min) and washed twice in phosphate-buffered saline. Subsequently, the cells were incubated in 100 mM potassium chloride (KCl), 5 mM magnesium chloride ($MgCl_2$), 1 mM phenylmethylsulphonyl fluoride and 50 mM HEPES–KOH (pH 7.4) for 1 h at 0°C, in order to allow the cells to swell. Then the cells were ultrasonicated at 20% of the maximum power of an MSE sonicator (Soniprep 150) for three bursts of 15 s. The suspension was centrifuged at 1500 ***g*** for 10 min. The post-nuclear supernatant was layered on top of a 46% sucrose cushion. After centrifugation at 100 000 ***g*** for 60 min the interface was removed and washed in 100 mM KC1–50 mM HEPES–5 mM $MgCl_2$ buffer (pH 7.4). The final membrane preparations were stored at –80°C at a protein concentration of ~ 4 mg ml$^{-1}$. The enrichment of $Na^+K^+$ATPase was about fivefold (Heijn et al, 1997).

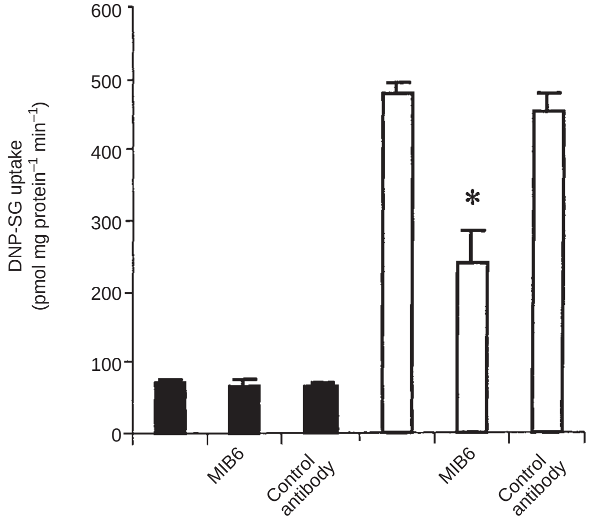

**Figure 1** ATP-dependent uptake of DNP-SG into $GLC_4$ and $GLC_4$/ADR membrane vesicles and its inhibition by the MIB6 antibody. Plasma membranes from $GLC_4$ cells (black bars) and $GLC_4$/ADR cells (white bars) were incubated with 10 nM DNP-SG in the absence or presence of the MIB6 antibody (MIB6) (10 µg ml$^{-1}$) or an MRP1 control antibody (10 µg ml$^{-1}$). Incubation time was 15 s. In the absence of ATP the uptake rate into $GLC_4$/ADR was comparable to $GLC_4$. Data are means ± s.e.m. ($n$ = 3). * indicates $P$-values < 0.05 compared to control (without MIB6) (Student's $t$-test)

### Uptake of substrates into vesicles

Uptake of radiolabelled substrates into vesicles was measured by rapid filtration as previously described (Heijn et al, 1997). Vesicles were incubated in KCl–HEPES buffer (pH 7.4) at 37°C (protein concentration ~ 0.25 mg ml$^{-1}$), in the presence of 10 mM $MgCl_2$ with or without 1 mM ATP and tritium-labelled DNP-SG. The reaction was stopped by adding ice-cold KCl–HEPES buffer. After rapid filtration, the filters were washed twice with KCl–HEPES buffer. The radioactivity on the filters was measured by liquid scintillation counting.

### ATPase activity determinations

To measure the ATPase activity in plasma membrane fractions we used a colourimetric method to estimate amounts of inorganic phosphate (Lanzetta et al, 1979) with some modifications. Plasma membranes were incubated for 1 h in KCl–HEPES buffer (pH 7.4) at 37°C, in the presence of 1 mM EGTA, 1 mM sodium azide and 0.1 mM ouabain, with or without 1 mM ATP. The reaction was linear during this period of time as shown before (Hooijberg et al, 1997). Reactions were stopped by addition of the colour reagent (0.034% w/v malachite green base, 1.05% w/v ammonium molybdate and 0.025% v/v Triton N101). After 1 min, sodium citrate was added to a final concentration of 3.6% (w/v), in order to

© 1999 Cancer Research Campaign

**Table 1** Flow cytometric analysis of MIB6 interaction with MRP1

| Cell line | Ratio mrpr1/lmr 94 | Ratio resistant/parent | Ratio MIB6/IgM | Ratio resistant/parent |
|---|---|---|---|---|
| $GLC_4$ | 2.8 ± 0.4 | 9.6 ± 3.2 | 5.5 ± 1.2 | 8.9 ± 2.6 |
| $GLC_4$/ADR | 27.0± 8.0 | | 49.0 ± 9.7 | |
| HL60 | 1.9 ± 0.2 | 4.0 ± 0.9 | 3.9 ± 1.0 | 4.1 ± 1.2 |
| HL60/ADR | 7.6 ± 0.4 | | 16.0 ± 2.5 | |
| SW1573 | 2.7 ± 0.8 | 3.0 ± 1.3 | 7.8 ± 5.4 | 2.6 ± 0.9 |
| S1(MRP) | 8.2 ± 2.5 | | 20.0 ± 1.5 | |

Numbers are ratios of mean fluorescence intensities derived from FACS histograms. Antibodies used are MRPr1 (0.35 μg $ml^{-1}$) and irrelevant lmr94 (both IgG2a) (Flens et al, 1994), and MIB6 (2 μg $ml^{-1}$) and irrelevant IgM antibody. $GLC_4$/ADR, HL60/ADR overexpress MRP1(Krishnamachary et al, 1994; Kool et al, 1997). S1(MRP) is a stable MRP1-transfectant of SW1573 (Zaman et al, 1995).

**Table 2** Effect of (iso)flavonoids on the accumulation of daunorubicin in $GLC_4$ cells and $GLC_4$/ADR cells

| | $GLC_4$ | $GLC_4$/ADR |
|---|---|---|
| Control | 100 ± 0 | 100 ± 0 |
| Genistein | 107 ± 7 | 354[a] ± 17 |
| Genistin | 101 ± 3 | 96 ± 4 |
| Kaempferol | 92 ± 7 | 190[a] ± 38 |
| Kaempferol-3 glucose | 88[a] ± 5 | 76[a] ± 8 |
| Apigenin | 91 ± 3 | 220[a] ± 45 |
| Apigetrin | 70[a] ± 11 | 57[a] ± 20 |
| Flavopiridol | 104 ± 2 | 242[a] ± 62 |

Table gives mean percentages of control (set at 100%) ± s.e.m. ($n$ = 3). Concentration (iso)flavonoid was 50 μM. [a]Significantly different from control, $P < 0.05$ (Student's $t$-test).

prevent ATP hydrolysis after having stopped the enzymatic reaction. Light absorption was measured in an enzyme-linked immunosorbent assay (ELISA) reader at a wavelength of 595 nm. No ATP hydrolysis was seen without the addition of membrane vesicles.

### MIB6 antibody and FACS analysis

The preparation and selection of the antibody MIB6 against the amino acid position 192–360 and 986–1204 of MRP1 will be described in detail elsewhere (Scheffer et al, in preparation). In short, anti-MRP1 antibodies were tested for their capacity to inhibit DNP-SG transport into $GLC_4$/ADR inside-out plasma membrane vesicles. One monoclonal antibody with potent inhibitory action was selected and named MRP1 Internal Binding 6 (MIB6). The isotype of MIB6 was IgM. The specificity of MIB6 for MRP1 was investigated in a FACS experiment using MRP1-transfected cells and several couples of MRP1 overexpressing cell lines and their parental cell lines. $GLC_4$/ADR cells and HL60/ADR cells overexpressed MRP1, but not MRP2–MRP5 (Kool et al, 1997). Cells were permeabilized with Becton Dickinson Lysing Solution containing 0.1% saponin (15 min, room temperature). The earlier described MRPr1 antibody against MRP1 (Flens et al, 1994) was used as a control for MRP1 expression, the lmr94 antibody (IgG2a) (Flens et al, 1994) and a mouse IgM antibody were used as negative controls. Rabbit–anti-mouse–FITC (fluorescein isothiocyanate) or rabbit–anti-rat–FITC were used as secondary antibody. The fluorescence was measured using a FACScalibur flow cytometer.

### Cellular drug accumulation

The steady-state cellular drug accumulation of [$^3$H]DNR was performed as described earlier (Broxterman et al, 1988). The assay was started by addition of 0.5 μM tritium-labelled DNR in the presence or absence of a modulator, which was added to the cells 5 min before DNR. Cells were incubated for 75 min at 37°C, followed by washing in ice-cold PBS. The amount of radioactivity was determined by scintillation counting.

### Growth inhibition assays

Cells were cultured in either flat-bottom 96-well plates or 24-well plates. One day after plating drugs were added at different concentrations. After 3 days of drug exposure, cells cultured in 24-well plates were counted using a micro-cellcounter. For cells cultured in 96-well plates a sulphorhodamine B (SRB) assay was performed (Skehan et al, 1990).

### RESULTS

### MIB6 is a specific antibody against MRP1

In order to produce a reagent able to determine the MRP1 specificity of drug-induced and (iso)flavonoid-induced effects, monoclonal antibodies were raised against MRP1 fusion proteins and selected for their ability to inhibit the ATP-dependent uptake of DNP-SG into plasma membrane vesicles from MRP1 overexpressing $GLC_4$/ADR cells. Of 19 MRP1-antibodies tested, only one inhibited uptake of DNP-SG. This monoclonal antibody was named MRP1 Internal Binding 6 (MIB6). Figure 1 shows the effect of MIB6 and one of the other tested MRP1 antibodies on the ATP-dependent uptake of DNP-SG into plasma membrane vesicles prepared from $GLC_4$/ADR cells and parental $GLC_4$ cells. The uptake of DNP-SG into $GLC_4$/ADR vesicles was 490 ± 7 pmol mg $protein^{-1}$ $min^{-1}$, using 100 nM DNP-SG. In the presence of MIB6 the uptake was decreased to about 50% of the uptake in the absence of MIB6. MIB6 had no effect on the uptake of DNP-SG into $GLC_4$ vesicles.

We further tested the specificity of the MIB6 antibody for MRP1 in several experiments that will be described in detail elsewhere

© 1999 Cancer Research Campaign

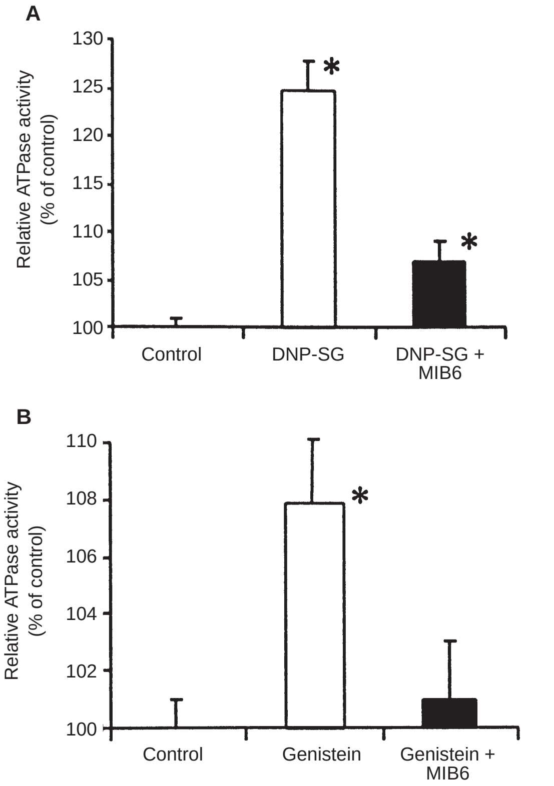

**Figure 2** Inhibition of the ATPase activity of $GLC_4$/ADR membranes by MIB6. (**A**) ATPase activity induced by 10 μM DNP-SG. (**B**) ATPase activity induced by 50 μM genistein. Concentration MIB6 was 10 μg $ml^{-1}$. Data are means ± s.e.m. ($n$ = 3). * $P < 0.05$ compared to control (Student's *t*-test)

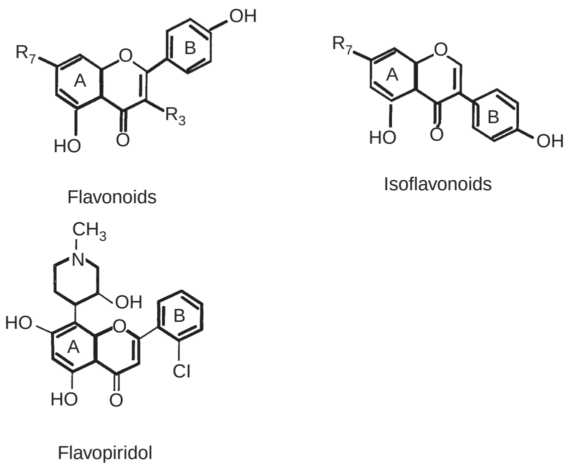

**Figure 3** Structure of (iso)flavonoids used in this study. Rings of the basic structure referred to in the Discussion are indicated with A or B. Flavonoids used were: apigenin (R3 = H; R7=OH), apigetrin (R3 = H; R7=glucose), kaempferol (R3=OH; R7=OH), kaempferol-3-glucose (R3 = glucose; R7 = OH) and flavopiridol. Isoflavonoids used were: genistein (R7 = OH) and genistin (R7 = glucose)

(Scheffer et al, in preparation). Here we show the MRP1 specificity of MIB6 as analysed by flow cytometry using three different pairs of parent and MRP1 overexpressing cells (Table 1). For comparison we used the established anti-MRP1 antibody MRPr1 (Flens et al, 1994). Table 1 shows the comparison between the fluorescence signals of MIB6-labelled cells and MRPr1-labelled cells. It can be seen that the ratios of the staining of MRP1 overexpressing cells to parent cells were very similar for both antibodies.

**Table 3** Cytotoxicity of (iso)flavonoids for different MRP1 overexpressing and parent cell lines

| | $IC_{50}$ value | | |
|---|---|---|---|
| **Cell** | **Flavopiridol** | **Genistein** | **Kaempferol** |
| $GLC_4$ | 45 ± 5 nM[a] | 12 ± 3 μM[b] | 25 ± 9 μM[b] |
| $GLC_4$/ADR | 128 ± 16 nM[a] | 13 ± 7 μM[b] | 11 ± 4 μM[b] |
| $GLC_4$[d] | > 1000 nM[a] | ND | ND |
| $GLC_4$/ADR[d] | > 1000 nM[a] | ND | ND |
| SW1573 | 85 ± 4 nM[c] | 10 ± 3 μM[c] | 29 ± 5 μM[c] |
| S1(MRP) | 130 ± 24 nM[c] | 9 ± 3 μM[c] | 38 ± 9 μM[c] |
| S1(1.1) | 93 ± 30 nM[c] | 9 ± 1 μM[c] | 43 ± 12 μM[c] |

[a]Mean ± s.e.m. ($n$ = 3). [b]Mean ± s.e.m. ($n$ = 5). [c]Mean ± s.e.m. ($n$ = 4). [d]Incubation of 1 h, followed by 71-h drug-free period. ND, not determined.

### Inhibition of MRP1 ATPase activity by MIB6

The capacity of MIB6 to inhibit DNP-SG-induced stimulation of the ATPase activity of membranes from $GLC_4$/ADR was measured. The basal ATPase activity of $GLC_4$/ADR membranes was stimulated by 10 μM DNP-SG to 4.5 ± 0.5 nmol mg $protein^{-1}$ $min^{-1}$ above the control. This was a stimulation of 125% compared to the control (Figure 2A). In the presence of MIB6, however, the induction of the ATPase activity of $GLC_4$/ADR membranes by DNP-SG was significantly lower (107% of control levels).

Next, we investigated the effect of MIB6 on the stimulation of the ATPase activity of $GLC_4$/ADR membranes by an MRP1 modulator, the isoflavonoid genistein (Figure 2B). The significant ATPase stimulation by 50 μM genistein to 108% of the control was almost completely inhibited in the presence of MIB6.

### Dose-dependent induction of MRP1 ATPase activity by (iso)flavonoids

Since we have shown that genistein induces ATPase activity of MRP1, we tested the dose-dependency of several (iso)flavonoids on the MRP1 ATPase activity of $GLC_4$/ADR membranes. Because many naturally occurring (iso)flavonoids are glycosylated (Kuehnau, 1976), we also investigated the effect of a glucose substitution on the (iso)flavonoid molecules on their interaction with MRP1.

$GLC_4$/ADR plasma membranes were incubated with one of the (iso)flavonoids genistein, genistin (= genistein-7-glucose), kaempferol, kaempferol-3-glucose, apigenin, or apigetrin (= apigenin-7-glucose) (see Figures 3 and 4). None of the tested (iso)flavonoids affected the ATPase activity of parental $GLC_4$ membranes (data not shown). The ATPase activity of $GLC_4$/ADR membranes was induced significantly by apigenin, kaempferol and genistein at concentrations as low as 1 μM and higher. Only for kaempferol, at 4 and 8 μM no significant effect was observed, for which we do not have an explanation so far. In contrast to the ATPase stimulation by the non-glycosylated compounds, the glycosylated (iso)flavonoids apigetrin, kaempferol-3-glucose and

© 1999 Cancer Research Campaign

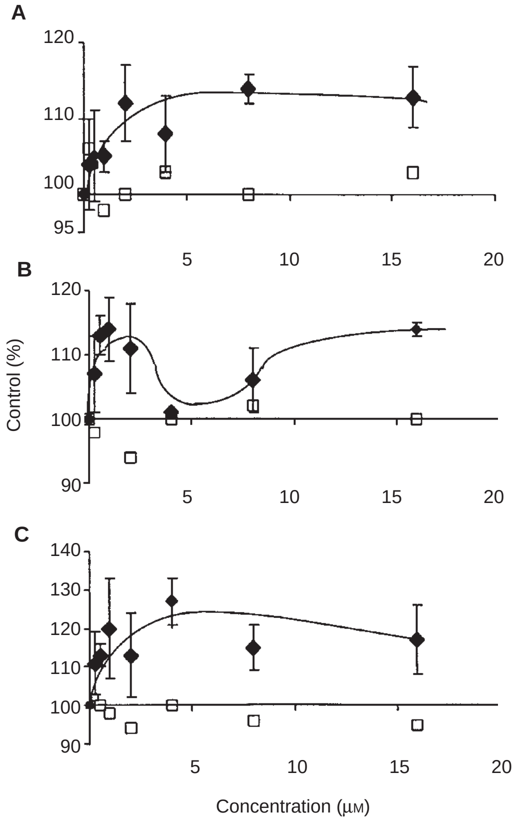

**Figure 4** Effect of (iso)flavonoids and glycosylated (iso)flavonoids on MRP1 associated ATPase activity. Data are means of at least three different experiments. Error bars are included if *P*-values < 0.05 compared to control (Student's *t*-test). Lines given are fitted (least squares method). (**A**) Induction by apigenin (filled symbols) and apigetrin (open symbols). (**B**) Induction by kaempferol (filled symbols) and kaempferol-3-glucose (open symbols). (**C**) Induction by genistein (filled symbols) and genistin (open symbols)

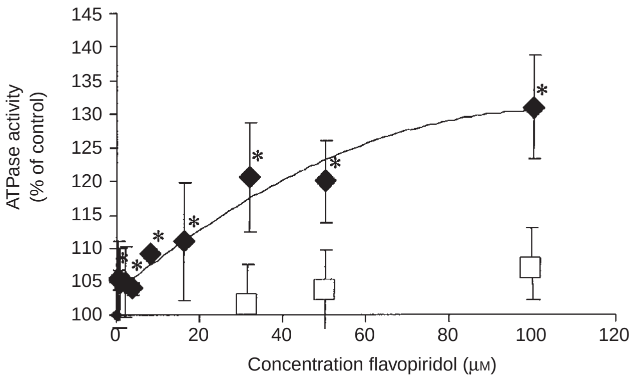

**Figure 5** Effect of flavopiridol on the MRP1-associated ATPase activity. Tested concentrations of flavopiridol were: 0 μM (control), 0.5, 1, 2, 4, 8, 16, 32, 50 and 100 μM (filled symbols). At 32 μM, 50 μM and 100 μM the effect of the MIB6 antibody (10 μg $ml^{-1}$) is shown (open symbols). Data are means of three independent experiments. * indicates *P*-values < 0.05 compared to control (Student's *t*-test). The line was fitted with a least square method genistin did not induce ATPase activity in the concentration range tested (Figure 4).

We were particularly interested in the MRP1-interaction of the synthetic anticancer agent flavopiridol. Flavopiridol stimulated the MRP1 ATPase activity at concentrations of 1 μM and higher, as shown in Figure 5. The MIB6 antibody inhibited this flavopiridol-induced ATPase activity almost completely (Figure 5).

Since it has been demonstrated that several uncharged and basic compounds can be transported by MRP1 only in the presence of millimolar concentrations of reduced glutathione (GSH) (Loe et al, 1997, 1998), we studied the effect of GSH on (iso)flavonoid-induced MRP1 ATPase activity. In the presence of GSH (0.5 mM or 5 mM) no synergistic stimulation of ATPase activity could be observed for flavopiridol, as well as other flavonoids (data not shown).

### Effect of flavopiridol and other (iso)flavonoids on the accumulation of DNR in MRP1-overexpressing cells

Earlier reports described the modulation of MRP1-mediated drug transport out of cells by high concentrations of genistein (Versantvoort et al, 1994). We studied whether flavopiridol and other (iso)flavonoids also affected cellular drug accumulation and whether this paralleled the described effects on MRP1 ATPase activity. The effect of a moderate concentration (50 μM) of (iso)flavonoids on the accumulation of DNR in $GLC_4$ and $GLC_4$/ADR was measured (Table 2). In the parental $GLC_4$ cells, no (iso)flavonoid effect on the DNR accumulation was observed. However, genistein, kaempferol, apigenin and flavopiridol significantly increased the accumulation of DNR in $GLC_4$/ADR cells. In contrast, the glycosylated compounds genistin, kaempferol-3-glucose and apigetrin did not increase accumulation of DNR in $GLC_4$/ADR cells. A small, but significant decrease of the DNR accumulation was seen in both cell lines for kaempferol-3-glucose and apigetrin, which points to a non-MRP1 related effect.

Since flavopiridol is currently being tested as an anticancer agent at low μM doses (Senderowicz et al, 1998; Stinson et al, 1998), we were particularly interested whether flavopiridol was able to modulate MRP1 function at much lower concentrations as well. We therefore tested a wider range of concentration flavopiridol from 100 nM to 50 μM and compared its effect on cellular DNR accumulation with that of genistein. Figure 6 gives the modulation of the accumulation of DNR in $GLC_4$/ADR cells by both compounds at concentrations lower than 10 μM. Already at the lowest concentration tested (100 nM) we observed a significant increase of DNR accumulation by flavopiridol. In contrast, low micromolar concentrations of genistein (and other flavonoids, data not shown) did not modulate the cellular accumulation of DNR in these cells.

### Growth inhibition by (iso)flavonoids of MRP1 overexpressing cells compared to drug-sensitive cells

We wanted to obtain information on the potential impact of MRP1 expression on the growth inhibitory effects of flavopiridol, genistein and kaempferol. For that, we tested these compounds on $GLC_4$ and $GLC_4$/ADR cells, SW1573, their *MRP-1* transfectant S1(MRP1) and their *MDR1* transfectant S1(1.1) (Zaman et al, 1995) (Table 3). First, it can be seen that flavopiridol is about 100-fold more potent compared to both other compounds. In addition, we observed resistance against flavopiridol in the $GLC_4$/ADR cells (2.8 times resistant compared to $GLC_4$) and in S1(MRP) cells (1.5 times resistant compared to SW1573). Simultaneous incubation

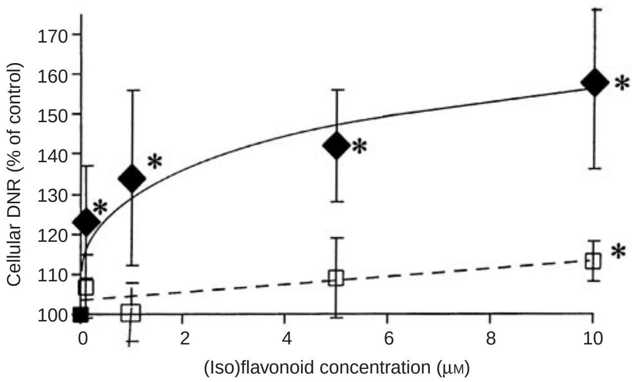

**Figure 6** Effect of flavopiridol (closed symbols) and genistein (open symbols) on daunorubicin accumulation in $GLC_4$/ADR cells. Data are means ± s.e.m. ($n$ = 3). The tested concentrations were: 0 μM (control), 0.1 μM, 1 μM, 5 μM and 10 μM. All tested flavopiridol concentrations and 10 μM genistein gave a significant difference (*) from the control ($P$ < 0.05, Student's $t$-test). Lines given are fitted (least square method)

with the established MRP1 inhibitor probenecid (Feller et al, 1995) (0.5 mM) did not alter the cytotoxic effect of flavopiridol on $GLC_4$ cells and $GLC_4$/ADR cells (data not shown). Short exposures (1 h) of flavopiridol to $GLC_4$ cells and $GLC_4$/ADR cells, followed by a drug-free culture period (71 h) resulted in $IC_{50}$ values above 1 μM for both cell lines. No resistance against genistein and kaempferol (72-h exposures) could be observed. Also, the *MDR1*-transfected S1(1.1) cells showed no resistance against either of the (iso)flavonoids.

### DISCUSSION

The multidrug resistance protein MRP1 seems to be expressed in all human cancers (Nooter et al, 1995). High levels of MRP1 expression have been found in chronic lymphocytic leukaemia, oesophagus squamous cell carcinoma and non-small-cell lung cancer (Nooter et al, 1995). Modulation of MRP1-mediated drug transport may be one way to improve the efficiency of chemotherapeutic treatment of cancer. One class of chemicals that has been reported to interact with MRP1 is the group of (iso)flavonoids (Versantvoort et al, 1993; Hooijberg et al, 1997). The interest in these compounds exceeds their use as MDR modulators, because of their broad spectrum of biochemical activities and their occurrence in our daily food as metabolic products of plants. Although in the past, several estimations have been made of the daily human intake of (iso)flavonoids (Kuehnau, 1976) these data are still a matter of dispute, due to lack of sensitivity of the analytical methods (Hollman et al, 1995). Recent reports showed that the synthetic flavonoid flavopiridol could reach steady-state plasma concentrations in humans of 200–300 nM during continuous infusion (Senderowicz et al, 1998; Stinson et al, 1998).

A number of biochemical activities have been described for some (iso)flavonoids, which relate to their effects on tumour cell proliferation. These biochemical activities include inhibition of phosphotyrosine kinases (Akiyama et al, 1987) and inhibition of topoisomerase II (Markovits et al, 1989). But also inhibition of angiogenesis has been described (Fotsis et al, 1993). Finally, certain (iso)flavonoids, in particular the synthetic flavonoid flavopiridol, have been reported to be specific inhibitors of cyclin-dependent kinases (Carlson et al, 1996; Sedlacek, 1996). In this respect, flavopiridol is 250-fold more potent compared to genistein and quercetin (Losiewicz et al, 1996). Therefore, flavopiridol is now the first potent cyclin-dependent kinase inhibitor to undergo clinical trials as an antineoplastic agent (Senderowicz et al, 1998; Stinson et al, 1998).

In this study we have performed an analysis of the effects of flavopiridol on MRP1 activity, and compared it with some of the typical members of the large group of natural (iso)flavonoids. Based on an earlier study (Hooijberg et al, 1997) we knew that flavopiridol and several natural (iso)flavonoids stimulate the ATPase activity of MRP1 containing $GLC_4$/ADR plasma membranes at relatively high micromolar concentrations. Two important considerations made us decide to study here the specific interaction of low micromolar concentrations of the (iso)flavonoids on MRP1 activity. First, in the light of the common intake with food, many (iso)flavonoids and their metabolites are present in low concentrations in human plasma (Hollman et al, 1997). Thus, the possibility that low plasma concentrations of many inhibitory compounds on MRP1 activity may act in an additive way must be considered. This has been described previously for Pgp modulators (Lyumbimov et al, 1995). Secondly, in particular with regard to flavopiridol, current clinical trials have achieved maximum tolerated doses of approximately 200–300 nM with a continuous infusion scheme (Senderowicz et al, 1998; Stinson et al, 1998).

In this study we have used plasma membrane vesicles from drug-resistant MRP1 overexpressing $GLC_4$/ADR cells to investigate MRP1-related ATPase activity and drug transport. As a tool to determine the MRP1 specificity of the (iso)flavonoid effects on transport and ATPase activity we have used a new monoclonal antibody against MRP1. This antibody, MIB6, supposedly binds to MRP1 at an internal epitope in intact membranes, since we observed interaction of this antibody with MRP1 in flow cytometry after membrane permeabilization and in immunoprecipitation experiments, but not on Western blots. Our results show that the ATP-dependent uptake of the MRP1 substrate DNP-SG in $GLC_4$/ADR plasma membrane vesicles, as well as the ATPase activity of $GLC_4$/ADR membranes induced by DNP-SG or (iso)flavonoids could be inhibited by MIB6.

We showed that several (iso)flavonoids, among which flavopiridol, stimulated MRP1 ATPase activity at low micromolar concentrations. Since this stimulation of ATPase activity was inhibited by MIB6, it was MRP1 specific. Stimulation of MRP1 ATPase activity has also been described by Chang et al (1997). The latter authors showed a rather small ATPase stimulation of semi-purified MRP1 by the substrates daunorubicin and doxorubicin. Addition of reduced GSH to these compounds resulted in an apparantly synergistic increase of ATPase stimulation. Besides, recent reports by Loe et al (1997, 1998) showed a GSH-dependent MRP1-mediated transport of basic, uncharged compounds. In our study, however, simultaneous incubation with GSH did not result in a synergistic increase of flavonoid-induced MRP1 ATPase activity.

Although much interest is directed towards non-glycosylated (iso)flavonoids, glycosylated flavonoids are in fact the most abundant species present in plants (Kuehnau, 1976). It was believed that these compounds could not be taken up in the intestine before deglycosylation by the intestinal flora (Bokkenheuser et al, 1987). However, recently it has been shown that glycosides of (iso)flavonoids can be detected in human plasma (Hollman et al, 1995, 1997). Therefore, we also explored the effects of glycosylated (iso)flavonoids on MRP1 activity. The glycosylated

© 1999 Cancer Research Campaign

(iso)flavonoids tested appeared unable to increase DNR accumulation in MRP1-overexpressing tumour cells, compared to the parent cells. This lack of enhancing effect of the glycosylated (iso)flavonoids on cellular DNR accumulation cannot be explained exclusively by a slow membrane passage of these compounds, since also the ATPase activity of MRP1 containing inside-out plasma membrane vesicles was not affected by the glycosylated (iso)flavonoids. Together, these observations concerning glycosylated (iso)flavonoids and their aglycones point to intrinsic differences in interaction with MRP1 between both classes of (iso)flavonoids.

When the tested compounds are examined in detail (Figure 3) it appears that the presence of a glucose moiety on position R7 of the A-ring of the (iso)flavonoid prevents the interaction with MRP1. However, since kaempferol-3-glucose also lacks an effect, it does not seem to be simply steric hindrance or the loss of two hydroxyl groups on the A-ring that is the cause. Moreover, the presence of the hydroxy-methyl-piperidinyl group on the A-ring of flavopiridol does not prevent MRP1 ATPase stimulation. Thus, not simply any substitution on this ring abolishes activity. Clearly, more extensive structure–activity analysis has to be done in order to define the exact structural requirements for (iso)flavonoid–MRP1 interaction. Also, it should be considered that effects on ATPase activity are not necessarily paralleled by similar effects on transport.

An interesting aspect of this study with respect to experimental cancer treatment is the fact that flavopiridol seems to be more potent as an inhibitor of MRP1-mediated drug transport than the other tested (iso)flavonoids (Versantvoort et al, 1993; present study). Natural non-glycosylated (iso)flavonoids were able to increase cellular DNR accumulation, only when present at high micromolar concentrations. However, a concentration as low as 100 nM flavopiridol affected DNR accumulation in MRP1-overexpressing cells. This concentration was lower than can be reached in human plasma during continuous infusion (Senderowicz et al, 1998; Stinson et al, 1998).

Since flavopiridol is proposed and tested not only as a single agent, but also in combination treatment with various anticancer drugs (Bible et al, 1997), our present data suggest that in future development of therapies with flavopiridol one should consider the interactions of this compound with MRP1 and its possible potentiation of MRP1 substrates, such as daunorubicin, vincristine and etoposide. On the other hand, a somewhat decreased activity of flavopiridol as single agent against MRP1-overexpressing tumours might be anticipated from the cross-resistance data presented in this study.

### ACKNOWLEDGEMENT

This study was supported by the Dutch Cancer Society (Grant KWF-VU-95-933).

### REFERENCES

Akiyama T, Ishida J, Nakagawa S, Ogawara H, Watanabe SI, Itoh N, Shibuya M and Fukami YJ (1987) Genistein, a specific inhibitor of tyrosine-specific protein kinases. *J Biol Chem* **262**: 5592–5595

Bible KC and Kaufmann SH (1997) Cytotoxic synergy between flavopiridol (NSC 649890, L86-8275) and various antineoplastic agents: the importance of sequence of administration. *Cancer Res* **57**: 3375–3380

Bokkenheuser VD, Shackleton CH and Winter J (1987) Hydrolysis of dietary flavonoid glycosides by strains of intestinal bacteroides from humans. *Biochem J* **248**: 953–956

Bors W and Saran M (1987) Radical scavenging by flavonoid antioxidants. *Free Rad Res Commun* **2**: 289–294

Broxterman HJ, Kuiper CM, Schuurhuis GJ, Tsuruo T, Pinedo HM and Lankelma J (1988) Increase of daunorubicin and vincristine accumulation in multidrug resistant human ovarian carcinoma cells by a monoclonal antibody reacting with P-glycoprotein. *Biochem Pharmacol* **37**: 2389–2393

Broxterman HJ, Giaccone G and Lankelma J (1995) Multidrug resistance proteins and other drug transport-related resistance to natural product agents. *Curr Opin Oncol* **7**: 532–540

Butler J, Spielberg SP and Schulman JD (1976) Reduction of disulphide containing amines, amino acids, and small peptides. *Anal Biochem* **75**: 674–675

Carlson BA, Dubay MM, Sausville EA, Brizuela L and Worland PJ (1996) Flavopiridol induces G1 arrest with inhibition of cyclin-dependent kinase (CDK)2 and CDK4 in human breast carcinoma cells. *Cancer Res* **56**: 2973–2978

Chang X, Hou Y and Riordan J (1997) ATPase activity of purified multidrug resistance-associated protein. *J Biol Chem* **272**: 30962–30968

Cole SPC and Deeley RG (1998) Multidrug resistance mediated by the ATP-binding cassette transporter protein MRP. *Bioessays* **20**: 931–940

Cole SPC, Bhardwaj G, Gerlach JH, Mackie JE, Grant CE, Almquist KC, Stewar AJ, Kurz EU, Duncan AM and Deeley RG (1992) Overexpression of a transporter gene in a multidrug-resistant human lung cancer cell line. *Science* **258**: 1650–1654

Cotelle N, Bernier JN, Henichart JP, Catteau JP, Gaydou E and Wallet JC (1992) Scavenger and antioxidant properties of ten synthetic flavones. *Free Rad Biol Med* **13**: 211–219

Critchfield JW, Welsh CJ, Phang JM and Yeh GC (1994) Modulation of adriamycin accumulation and efflux by flavonoids in HCT-15 colon cells. *Biochem Pharmacol* **48**: 1437–1445

Drees M, Dengler WA, Roth T, Abonte H, Mayo J, Malspeis L, Grever M, Sausville EA and Fiebig HH (1997) Flavopiridol (L86-8275): selective anti-tumour activity in vitro and activity in vivo for prostate carcinoma cells. *Clin Cancer Res* **3**: 273–279

Feller N, Broxterman HJ, Währer DCR and Pinedo HM (1995) ATP-dependent efflux of calcein by the multidrug resistance protein (MRP): no inhibition by intracellular glutathione depletion. *FEBS Letters* **368**: 385–388

Filgueira de Azevedo Jr W, Mueller-Dieckmann HJ, Schulze-Gahmen U, Worland PJ, Sausville E and Kim SH (1996) Structural basis for specificity and potency of a flavonoid inhibitor of human CDK2, a cell cycle kinase. *Proc Natl Acad Sci USA* **93**: 2735–2740

Flens MJ, Izquierdo MA, Scheffer GL, Fritz JM, Meijer CJLM, Scheper RJ and Zaman GJR (1994) Immunochemical detection of the multidrug resistance-associated protein MRP in human multidrug resistant tumour cells by monoclonal antibodies. *Cancer Res* **54**: 4557–4563

Fotsis T, Pepper MS, Adlercreutz H, Fleischmann G, Hase T, Montesano R and Schweigerer L (1993) Genistein, a dietary-derived inhibitor of in vitro angiogenesis. *Proc Natl Acad Sci USA* **90**: 2690–2694

Glossmann H, Presek P and Eigenbrot E (1981) Quercetin inhibits tyrosine phosphorylation by the cyclic nucleotide-dependent, transforming protein kinase, pp60src. *Naunyn-Schmiedeberg Arch Pharmakol* **317**: 100–102

Gottesman MM and Pastan I (1993) Biochemistry of multidrug resistance mediated by the multidrug transporter. *Annu Rev Biochem* **62**: 385–427

Gugler R, Leschik M and Dengler HJ (1975) Disposition of quercetin in man after single oral and intravenous doses. *Eur J Clin Pharmacol* **9**: 229–234

Heijn M, Oude Elferink RPJ and Jansen PLM (1992) ATP-dependent multispecific anion transport system in rat erythrocyte membrane vesicles. *Am J Physiol* **262**: 104–110

Heijn M, Hooijberg JH, Scheffer GL, Szabó G, Westerhoff HV and Lankelma J (1997) Anthracyclines modulate multidrug resistance protein (MRP) mediated organic anion transport. *Biochim Biophys Acta* **1326**: 12–22

Higgins CF (1992) ABC transporters: from micro-organisms to man. *Annu Rev Cell Biol* **8**: 67–113

Hoffmann J, Doppler W, Jakob A, Maly K, Posch K, Uberall F and Grunicke H (1988) Enhancement of the anti-proliferative effect of cis-diamminedichloroplatinum(II) and nitrogen mustard by inhibitors of protein kinase C. *Int J Cancer* **42**: 382–388

Hollman PCH, de Vries JH, van Leeuwen SD, Mengelers MJ and Katan MB (1995) Absorption of dietary quercetin glycosides and quercetin in healthy ileostomy volunteers. *Am J Clin Nutr* **62**: 1276–1282

Hollman PCH, van Trijp JMP, Buysman MNCP, vander Gaag MS, Mengelers MJB, de Vries JHM and Katan MB (1997) Relative bioavailability of the antioxidant flavonoid quercetin from various foods in man. *FEBS Lett* **418**: 152–156

Hooijberg JH, Broxterman HJ, Heijn M, Fles DLA, Lankelma J and Pinedo HM (1997) Modulation by (iso)flavonoids of the ATPase activity of the multidrug resistance protein. *FEBS Lett* **413**: 344–348

© 1999 Cancer Research Campaign 

Jedlitschky G, Leier I, Buchholz U, Center M and Keppler D (1994) ATP-dependent transport of glutathione S-conjugates by the multidrug resistance-associated protein. *Cancer Res* **54**: 4833–4836

Kool M, de Haas M, Scheffer GL, Scheper RJ, van Eijk MJT, Juijn JA, Baas F and Borst P (1997) Analysis of expression of cMOAT (MRP2), MRP3, MRP4, and MRP5, homologues of the multidrug resistance-associated protein gene (MRP1), in human cancer cell lines. *Cancer Res* **57**: 3537–3547

Krishnamachary N, Ma L, Zheng L, Safa AR and Center MS (1994) Analysis of MRP gene expression and function in HL60 cells isolated for resistance to adriamycin. *Oncol Res* **6**: 119–127

Kuehnau J (1976) The flavonoids. A class of semi-essential food components: their role in human nutrition. *World Rev Nutr Diet* **24**: 117–191

Lanzetta PA, Alvarez LJ, Reinach PS and Candia OA (1979) An improved assay for nanomole amounts of inorganic phosphate. *Anal Biochem* **100**: 95–97

Loe DW, Stewart RK, Massey TE, Deeley RG and Cole SPC (1997) ATP-dependent transport of aflatoxin B-1 and its glutathione conjugates by the product of the Multidrug Resistance Protein (MRP) gene. *Mol Pharmacol* **51**: 1034–1041

Loe DW, Deeley RG and Cole SPC (1998) Characterization of vincristine transport by the MRP 190 000 multidrug resistance protein (MRP) – evidence for cotransport with reduced glutathione. *Cancer Res* **58**: 5130–5136

Losiewicz MD, Carlson BA, Kaur G, Sausville EA and Worland PJ (1996) Potent inhibition of cdc2 kinase activity by the flavonoid L86-8275. *Biochem Biophys Res Commun* **201**: 589–595

Lyumbimov E, Lan LB, Pashinsky I, Ayesh S and Stein WD (1995) Saturation reversal of the multidrug pump using many reversers in low-dose combinations. *Anti Cancer Drugs* **6**: 727–735

Markovits J, Linassier C, Fossé P, Couprie J, Pierre J, Jacquemin-Sablon Saucier J.-M, Le Pecq J-B and Larsen AK (1989) Inhibitory effects of the tyrosine kinase inhibitor genistein on mammalian DNA topoisomerase II. *Cancer Res* **49**: 5111–5117

Müller M, Meijer C, Zaman GJR, Borst P, Scheper RJ, Mulder NH, de Vries EGE and Jansen PLM (1994) Overexpression of the gene encoding the multidrug resistance-associated protein results in increased ATP-dependent glutathione S-conjugate transport. *Proc Natl Acad Sci USA* **91**: 13033–13037

Nooter K, Westerman MA, Flens MJ, Zaman GJR, Scheper RJ, van Wingerden KE, Burger H, Oostrum R, Boersma T, Sonneveld P, Gratema JW, Kok T, Eggermont AMM, Bosman FT and Stoter G (1995) Expression of the multidrug resistance-associated protein (MRP) gene in human cancers. *Clin Cancer Res* **1**: 1301–1310

Sedlacek HH, Czech J, Naik R, Kaur G, Worland P, Losiewicz M, Parker B, Carlson B, Smith A, Senderowicz A and Sausville E (1996) Flavopiridol (L86 8275 NSC 649890): a new kinase inhibitor for tumor therapy. *Int J Oncol* **9**: 1143–1168

Senderowicz AM, Headlee D, Stinson SF, Lush RM, Kalil N, Villalba L, Hill K, Steinberg SM, Figg WD, Tompkins A, Arbuck SG and Sausville EA (1998) Phase I trial of continuous infusion of flavopiridol, a novel cyclin-dependent kinase inhibitor, in patients with refractory neoplasms. *J Clin Oncol* **16**: 2986–2999

Skehan P, Storeng R, Scudiero D, Monks A, McMahon J, Vistica D, Warren JT, Bokesch H, Kenney S, Boyd MR (1990) New colorimetric cytotoxicity assay for anticancer-drug screening. *J Natl Cancer Inst* **82**: 1107–1112

Srivastava AK (1985) Inhibition of phosphorylase kinase, and tyrosine protein kinase activities by quercetin. *Biochem Biophys Res Comm* **131**: 1–5

Stinson SF, Hill K, Siford TJ, Phillips LR, Daw TW (1998) Determination of flavopiridol (L86 8275; NSC 649890) in human plasma by reversed-phase liquid chromatography with electrochemical detection. *Cancer Chemoth Pharmacol* **42**: 261–265

Versantvoort CHM, Broxterman HJ, Pinedo HM, Feller N, Kuiper CM and Lankelma J (1992) Energy-dependent processes involved in reduced drug accumulation in multidrug resistant human lung cancer cell lines without P-glycoprotein expression. *Cancer Res* **52**: 17–23

Versantvoort CHM, Schuurhuis GJ, Pinedo HM, Eekman CA, Kuiper CM, Lankelma J and Broxterman HJ (1993) Genistein modulates the decreased drug accumulation in non-P-glycoprotein mediated multidrug resistant tumour cells. *Br J Cancer* **68**: 939–946

Versantvoort CHM, Broxterman HJ, Lankelma J, Feller N and Pinedo HM (1994) Saturation and inhibition of the ATP-dependent daunorubicin transport in an MRP overexpressing multidrug resistant human small cell lung cancer cell line. *Biochem Pharmacol* **48**: 1129–1136

Walgren RA, Walle UK and Walle T (1998) Transport of quercetin and its glycosides across human intestinal epithelial caco-2 cells. *Biochem Pharm* **55**: 1721–1727

Zaman GJR, Flens MJ, van Leusden MR, de Haas M, Mülder HS, Lankelma J, Pinedo HM, Scheper RJ, Baas F, Broxterman HJ and Borst P (1994) The human multidrug resistance-associated protein MRP is a plasma membrane drug-efflux pump. *Proc Natl Acad Sci USA* **91**: 8822–8826

Zaman GJ, Lankelma J, van Tellingen O, Beijnen J, Dekker H, Paulusma C, Oude Elferink RP, Baas F and Borst P (1995) Role of glutathione in the export of compounds from cells by the multidrug resistance-associated protein. *Proc Natl Acad Sci USA* **92**: 7690–7694

 *© 1999 Cancer Research Campaign*
